# Neurological Disorder Brain Model: A Lesson from Marine Worms (Annelida: Polychaeta)

**DOI:** 10.21315/mjms2019.26.6.2

**Published:** 2019-12-30

**Authors:** Mohd Ulul Ilmie Ahmad Nazri, Izwandy Idris, Othman Ross, Wan Iryani Wan Ismail

**Affiliations:** 1 Faculty of Science and Marine Environment, Universiti Malaysia Terengganu, Terengganu, Malaysia; 2 Institute of Oceanography and Environment, Universiti Malaysia Terengganu, Terengganu, Malaysia

**Keywords:** nervous system regeneration, marine polychaete, brain signalling pathway, neurodegenerative disease model, Alzheimer’s disease, Parkinson’s disease

## Abstract

The incidence of neurodegenerative diseases is directly proportional to age. The prevalence of non-communicable diseases, for example, Alzheimer’s and Parkinson’s, is expected to rise in the coming years. Understanding the etiopathology of these diseases is a crucial step that needs to be taken to develop drugs for their treatment. Animal models are being increasingly used to expand the knowledge and understanding on neurodegenerative diseases. Marine worms, known as polychaetes (phylum Annelida), which are abundantly and frequently found in benthic environments, possess a simple yet complete nervous system (including a true brain that is centralised and specialised) compared to other annelids. Hence, polychaetes can potentially be the next candidate for a nerve disease model. The ability to activate the entire nervous system regeneration (NSR) is among the remarkable features of many polychaetes species. However, the information on NSR in polychaetes and how it can potentially model neurodegenerative diseases in humans is still lacking. By exploring such studies, we may eventually be able to circumvent the developmental constraints that limit NSR in the human nervous system. This article is intended to briefly review responsible mechanisms and signalling pathways of NSR in marine polychaetes and to make a comparison with other established models of neurodegenerative disease.

## Introduction

The regeneration of body parts, such as appendages, muscles, hair, nails and cells, has been well studied both in the fundamental and applied sciences (1–2). However, the mechanism of the nervous system regeneration (NSR) is still poorly understood. Although considerable literature has been written on several regeneration models for neurodegenerative disease, including *Danio rerio* (zebrafish) for tissue regeneration (3), *Drosophila melanogaster* (fruit fly) for axon regeneration (4) and *Caenorhabditis elegans* (nematode) for neuronal and axon regeneration (5–6), the information on the whole nervous system, including the brain regeneration model, is lacking. For that reason, there is a need to search for a new model to represent the entire NSR. The model’s nervous system must comply with the human nervous system, that is, complete with a true brain and a centralised, specialised and developed nervous system, as well as being capable of regenerating. Polychaeta, in general, has the most developed nervous system and sensory organs compared to other classes in the phylum Annelida (7), which might be useful in comparison with the human nervous system. Remarkably, some polychaetes can regenerate the body upon injury (8). For instance, it is observed in genus *Diopatra* (Onuphidae) that some can regenerate both anteriorly and posteriorly, that is, *D. sugokai* (9), *D. dexiognatha* (10) and *D. neapolitana* (11). However, the use of polychaetes as a nervous system disease model has still not been realised. Nevertheless, many scientific works are still needed to discover the trends, patterns and factors crucial for NSR in polychaetes. Currently, the available information provides initial clues of the molecular target and signalling at the specific region of the nervous system that should be the reference to begin with. In the future, studies involving polychaetes will not only focus on their NSR capability, but on the application of NSR in finding a new cure for neurodegenerative diseases. Thus, this review will focus on the NRS and aim to highlight the potential of polychaetes to facilitate an understanding of the pathophysiology of neurodegenerative diseases.

## Polychaetes

**Figure 1:**
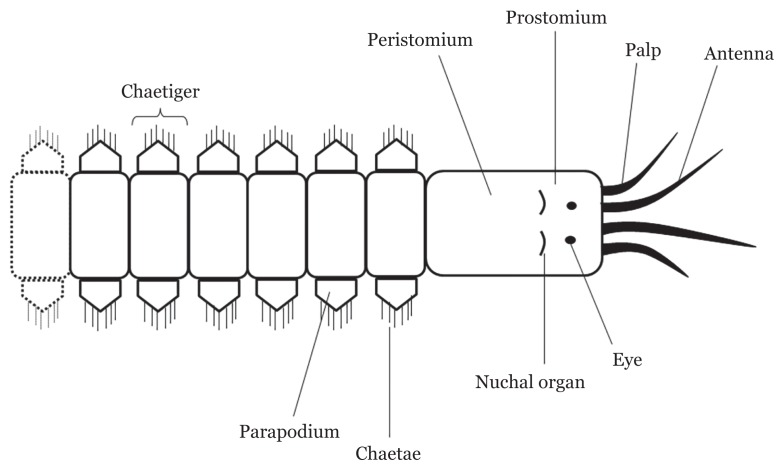
General outer anatomy of the anterior part of polychaete. Note that every chaetiger (segment) has parapodium with protruding chaetae use locomotion or defence

The polychaeta is the largest and most ancient class in the phylum Annelida, consisting of over 14,000 species that mainly inhabit marine environments (12). Polychaetes refer to segmented worms ranging from less than a millimetre to several metres in length (13), possessing little bristle-like structures, attached at the outermost part of the body, responsible for locomotion and defence (14). The bristles (chaetae), which are siliceous, chitinous and calcareous, are located at a pair of appendages called parapodia (singular: parapodium) existing at each segment (Figure 1) (15). They are normally grouped into sedentary or errantry forms (16). This type of adaptation reflects the nature of their habitats, as well as their lifestyle. Commonly, polychaetes are found burrowing in sediment on sands of beaches, freshwater and terrestrial and deep water, or they live in tubes either as a single occupant or in groups by forming calcareous reef structures. Nevertheless, some polychaetes species are pelagic or live as commensals or parasites (17).

## Polychaete Nervous System and Its Organisation

**Figure 2:**
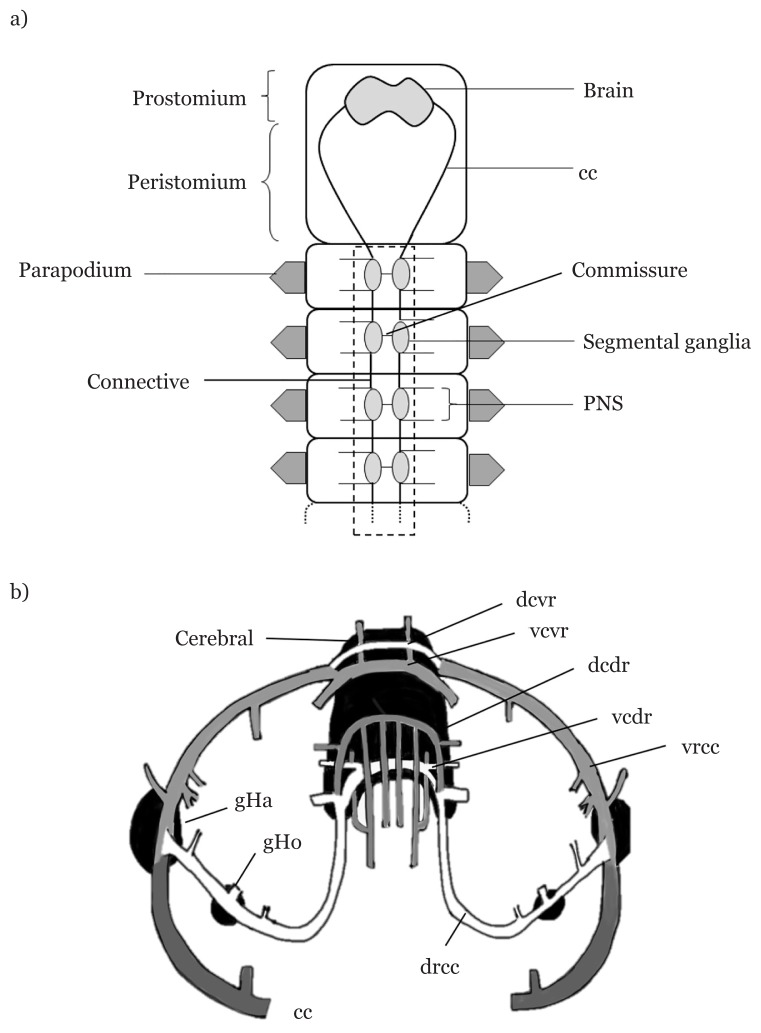
Schematic drawing of polychaete nervous system. a) The general organisation consists of brain, circumpharyngeal connectives symmetrically located and ladder-like VNC (perforated box). Aligned along the nerve cord is a pair of segmental ganglia connected by commissure and connected to other ganglia by connectives. b) The cephalic nerve organisation (22) consists of mainly nerves and ganglia Notes: drcc = dorsal root; vrcc = ventral root; dcdr and vcdr = dorsal and ventral commissures of dorsal root; dcvr and vcvr = dorsal and ventral commissures of ventral root; gHa = Hamaker’s ganglia; gHo = Homlgren’s ganglia; cc = circumpharyngeal connective

The nervous system for polychaete is considered to be a deeply conservative organ system (18). In addition, the nervous system varies among different polychaete families. The polychaete nervous system consists of the brain [cephalic nervous system (CpNS)], which is located in the prostomium region, the ventral nerve cord (VNC) and peripheral nervous system (PNS) [Figure 2a)]. The VNC forms a ladder-like pattern (19), consisting of a pair of segmental ganglia connected to the CpNS via circumpharyngeal connectives. The VNC can exist either as a single strand or double strand (20–21). Orrhage (22) proposed a synoptic illustration on CpNS that depicts the nerves innervating the prostomial appendages with a circumpharyngeal connective split symmetrically [Figure 2b)]. Furthermore, the ladder-like VNC consists of two ganglia in each segment linked by commissures. The ganglia are connected to other ganglia from the neighbouring segment via connectives (23). The part that completes the nervous system is the PNS, which is composed of the epidermal plexus, segmental nerves branching off from the connectives, and longitudinal nerves branching off from the brain (24).

The nerve organisation in the CpNS contains nerves and ganglia. They are arranged in such a way that they stack against each other through either the dorsal or ventral, and either the commissure or root. The main nerve is called the circumpharyngeal connective (cc), which is symmetrically arranged along the pharynx at the prostomium. These nerves then innervate to either the dorsal or ventral roots of the connectives (drcc and vrcc). There are two types of ganglions (Hamaker’s and Homlgren’s) in the CpNS, with different sizes and locations. Hamaker’s ganglia are located at the meeting points of drcc and vrcc, whereas Homlgren’s ganglia are located at drcc, lying further towards the cerebral [Figure 2b)]. Furthermore, each root will further innervate into dorsal or ventral commissures (dcdr, vcdr, dcvr and vcvr) to complete the cephalic nervous system in polychaetes, generally as summarised by Orrhage (22).

The information gathered from studies conducted on the characterisation of the polychaete’s nervous system could help us understand how the nervous system in polychaete and humans could be similar in a way that can represent diseases.

## Polychaete Nervous System versus Human Nervous System

**Figure 3:**
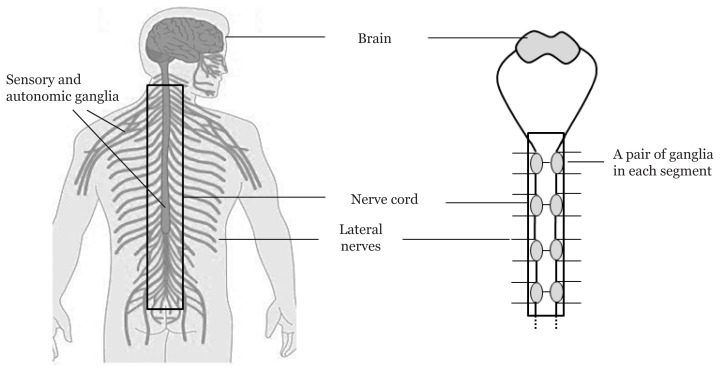
Comparison of human and polychaete nervous systems. In polychaete, brain is considered as a pair of ganglia as other pairs in each segment and responsible for the activities for the respective segments. Ganglia in human are located at various places and functioning in different roles

Having a bilateral symmetry nervous system similar to that of humans allows us to understand the signalling mechanism. Generally, a complete nervous system in both humans and polychaete consists of the brain, nerve/spinal cord, lateral nerves and neuronal cells (Figure 3). Other shared features are bundled neurons in axonal tracts, wrapped in glia and surrounded by circulating blood cells that carry out immune and phagocytic functions (4). In both organisms, there are structures called ganglia (singular: ganglion), which place a cluster of nerve cells or cell bodies, as well as dendritic structures. They are interconnected, forming a structure called plexus. In polychaetes, a pair of ganglia controls the actions in each body segment. The polychaete brain is also a ganglion that controls activities, mostly in the peristomium and prostomium regions (25).

In humans and other vertebrates, ganglia are categorised as sensory and autonomic ganglia. Sensory ganglia involve a branching of sensory endings to either the spinal cord or brain. Meanwhile, autonomic ganglia distribute at the sympathetic and parasympathetic nervous systems, being located near vertebral bodies and walls of organs (26). However, in the mammalian central nervous system, neurons display poor capability to regenerate upon damage. The ability to regenerate in polychaetes is fascinating because the process can be initiated even though the control centre is absent.

## Polychaetes with Regenerating Ability

Most polychaetes are capable of regenerating to some degree; the degree of regeneration varies widely across the taxon. Regeneration may take place from both the anterior and posterior segments or from either one of these regions. Some are capable of regenerating an entire individual from a single mid-body segment, as for example, in species from the Sabellidae, Chaetopteridae and Lumbriculidae (27–28). However, some species are not capable of regenerating from any side. For neurodegenerative diseases, the ability to regenerate the anterior part is the main focus. Table 1 presents the list of polychaete species capable of regenerating the anterior region as listed by Bely (29), with some additional species from recent publications (30–48).

## NSR Capability in Polychaetes

Research on polychaetes mainly involved fields such as taxonomy, evolution, behavioural assessment (49–50), pollution determination (51), ecology, aquaculture (52), natural product and physiology. However, detailed studies on the regeneration capability are still insufficient even though the capability to regenerate among polychaetes has long been recorded. We noted that the progress of regeneration studies is currently emerging; several studies are available on the pattern formation of the nervous system (35, 53), molecular data on genes or proteins that might be involved, and more species (54–55) that have been found to regenerate, but the initiation of NSR is still not clear.

In a species that can regenerate, successful regeneration might depend on some factors. One of the factors involves the amputation at certain chaetigers, such as *D. neapolitana*, which showed successful regeneration, whereas other chaetigers failed for unknown reasons (11). Furthermore, Weidhase et al. (33) documented the nervous system formation by performing immunohistochemistry on *Cirratulus* cf. *cirratus* while regenerating. The nervous system formation for anterior and posterior ends has also been observed in *Dorvillea bermudensis* through an immunohistochemical analysis (35). To date, the reports on the nervous system pattern in polychaete are tremendous and well understood, following the work done by Orrhage (22), who proposed a synoptic cephalic nervous system of polychaete [Figure 2b)].

## Polychaete as NSR Model

Model organisms are used in the laboratory with the assumption that the models act similarly or in a predictably different way compared to the organism of interest, for example, humans. However, the use of animals from higher taxa, such as rodents and primates, has been criticised from every angle, even though they show greater phyletic relatedness to humans. Apart from being difficult to handle, the psychological state of these animals might influence the result of the experiment (56). Moreover, the costs per individual and maintenance are so much higher as compared to most invertebrates (57). Hence, the search for a test subject from lower taxa that can show or predict the human response is increasingly progressing. Although the species-relatedness of humans and lower taxa animals is far apart, both taxa share a number of similarities. Species such as *D. melanogaster*, *C. elegans* and *D. rerio* dominate in genetic, cellular biology, physiological and developmental studies because these are inexpensive to maintain, easy to reproduce, have short generation times, and the tools for their genetic manipulation have been well described (58). Many of these species are subjected to large-scale early mutational screens. These aspects should be investigated in polychaetes as well because their nervous system is particularly amenable to such studies. This is due to its simplicity in terms of the number of neurons. Furthermore, the neurons are large and easy to identify and access. This is crucial for looking into the potential of polychaetes to become a reference subject and model that is without extreme complexity, yet complete and translatable that should be added to or used to complement other available models.

## The Underlying Mechanisms of Regeneration

The nervous system navigates very different conditions when it encounters regeneration compared to the one during development. However, some developmental growth and guidance cues might be maintained in adults, while others are lost or replaced by inhibitory factors (59). Regeneration is regulated by both intrinsic and extrinsic pathways. There are several proposed mechanisms of injury signalling, that is, calcium entry, electrical signals and changes in the trafficking of regeneration factors (59).

## Intracellular Regeneration Regulations

The regeneration process regulated by the intrinsic factors in polychaetes might involve several pathways that have already been studied in other organisms. Herewith, we list four intrinsic pathways involved when activated by injury or autotomy. Firstly, the Notch signalling occurs to inhibit regeneration from being processed, and the receptor (*lin-12* Notch) can be blocked by the γ-secretase inhibitor DAPT (N-[(3, 5-difluorophenyl) acetyl]-Lalnyl-2-phenyl] glycine-1, 1-dimethylethyl ester) to increase the regeneration (59). In a recent study, Notch has been reported to play no major role in neurogenesis in the larvae of a polychaete, *Platynereis dumerilii;* instead, impairing Notch signalling induces defects in chaetal sac formation (60). Secondly, calcium and cyclic adenosine monophosphate (cAMP) signalling will promote the axon regeneration due to increased intracellular calcium as a result of injury (61). The calcium enters the cell through the breached axonal membrane, which activates the voltage-gated calcium channels and is released from the intracellular stores (62). It has been shown that the calcium baseline can be reduced and regeneration can be impaired by treating with BAPTA (1, 2-bis(o-aminophenoxy) ethane N, N, N’, N’ tetraacetic acid).

**Figure 4:**
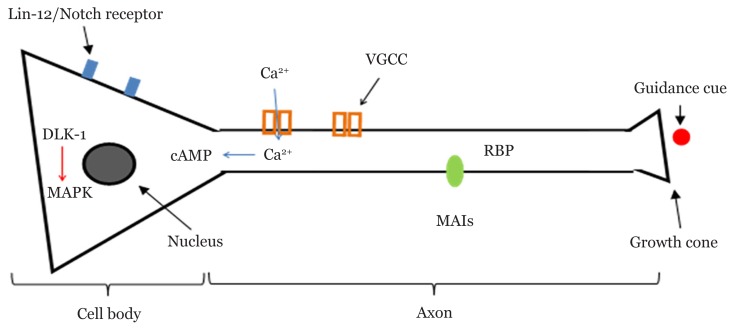
Summary of potential mechanisms for NSR in polychaetes. Intracellular regulations involve inhibition of Notch signal, Ca^2+^ influx through VGCC that phosphorylates cAMP and MAPK signalling. Extracellular regulations involve binding of MAI from oligodendrocyte to RNA binding protein (RBP), growth cone movement by a guidance cue and facilitation by ECM in cell migration, differentiation and proliferation. Notes: cAMP = cyclic adenosine monophosphate; DLK-1 = dual leucine zipper kinase; MAI = myelin-associate inhibitor; MAPK = mitogen-activated protein kinase; VGCC = voltage-gated calcium channel

The third pathway involves factors on the cell surface, which are channels, transporters and neurotransmitters. The injured neuron was shown to have a different action potential firing compared to the normal firing. This is probably due to the spread of neurotransmitter serotonin that is released by interneurons during the event (63). Serotonin promotes growth by affecting the cAMP pathways that lead to gene activation (64). Furthermore, the mutation that impairs the synthesis of another neurotransmitter called acetylcholine results in decreased regeneration (65). The next pathway is the mitogen-activated protein kinase (MAPK) signalling, which regulates proliferation, gene expression and so on. Yan et al. (66) have reported that the MAPK pathway is activated by injury. The RNAi screen in *unc-70*/β-spectrin mutants for genes affecting regeneration has identified *dlk-1* (gene for protein delta homolog) as a candidate regeneration gene (67). In addition, the overexpression of *dlk-1* increases regeneration (67). The mechanism is summarised in Figure 4.

## Extracellular Regeneration Regulations

The environment of the outer region of neurons or axons also plays some role in ensuring that the regeneration occurs. Extrinsic factors such as myelin and glial scar might potently inhibit regeneration in the nervous system. Myelin-associated inhibitors from oligodendrocytes happen to be the barriers to axonal growth after injury (68). However, a recent study has suggested other crucial factors in regeneration: axon guidance and elements in the extracellular matrix (ECM). Axon guidance is an important mechanism in the neural development by which the axon extends to reach out to a certain target. A growing axon has a structure called a growth cone that senses a guidance cue leading to a movement towards or away from the guidance cue due to intracellular signalling that happens inside the growth cone (69).

In the morphogenetic development of embryos and adults, the ECM plays a crucial role to ensure the stability of the tissue’s specific structures and functions (70). Other than that, the ECM controls the migration of neural crest cells and the growth of nerve endings (71–72). The basement membrane, a specific epithelial of the ECM, has been found to be involved in wound healing, scar formation and regeneration following injury, functioning in cell migration, differentiation and proliferation (73).

## Translation of the NSR to Neurodegenerative Disease Treatment

Generally, neurons cannot be reproduced, repaired or replaced (74). This condition occurs mostly in vertebrates, including humans. When the neuron is damaged due to injury or is worn out, a condition called neurodegeneration will take over. Neurodegenerative conditions are untreatable and debilitating due to a progressive degeneration or death of nerve cells. One of the diseases caused by neurodegeneration is Alzheimer’s, with dementia as a prominent symptom. Dementia is a condition where a person has difficulty thinking and remembering daily activities. Other symptoms, such as emotional and language problems, lack of motivation and memory loss, become worse as the person gets older (75).

Alzheimer’s disease is the most prevalent form of dementia, and it is estimated that the number of its sufferers is going to increase to 66 million in 2030 from 36 million in 2010 (75). In the human brain, it affects the hippocampus and influences learning and memory abilities. Even though these damages are not reversible in humans, understanding how polychaetes successfully regenerate their brain specifically may lead to various medical opportunities. In the field of regenerative medicine, researchers are working progressively to regrow, repair or replace the damaged cells by using therapeutic stem cells, tissue engineering and artificial organs. Maybe, after the mechanism of NSR is explored in detail in polychaetes, we could translate the capabilities to brain disease treatments in humans.

## Comparison of Other Nervous System Models with the Polychaete Model

Comparable models that are readily obtained and pay great attention to the nervous system are *D. rerio*, *D. melanogaster* and *C. elegans*, as stated earlier in this review. These models have been applied to represent disparate systems in humans to provide a better understanding of human-related issues. The prevailing popular application among the models is the study of regeneration since this ability is believed to be able to provide ways to cure neurodegenerative diseases (76). The attention now is directed towards an organism that would be able to reflect the NSR as a whole, including neurites, neurons, glial cells, axons or even dendritic spines. The next potential model also should be easy to handle, cost-favourable and reproducible. Table 2 displays a comparison between the three famous models and the potential model, polychaetes (77–81).

Another merit on experimentation using invertebrates is that no animal ethics approval is required except for the class Cephalopoda (82). However, concerns about using invertebrates in research are still being debated and need a rigorous professional standard care oversight (83). In addition, polychaetes are abundant, easy to handle and most of the time, not dangerous. Another critical factor that contributes to the suitability of polychaetes as a model is their ability to reproduce sexually and asexually. By understanding their habitat, we could also establish similar laboratory conditions in a more controlled environment. However, there is a lot more to be explored concerning the suitability of polychaetes to represent human body systems and physiology.

## Conclusion

Investigation of NSR in polychaetes that have the ability to rebuild their brains should be a new focus of neuromedicine. Subsequently, understanding the mechanisms of NSR will create possibilities for other findings. The application of various approaches ranging from tissue to molecular, that is immunohistochemistry, cell signalling, bioelectrical activity and gene expression, might potentially lead to the creation of a disease model, even though it is still far beyond our thinking how such organisms could benefit human beings in understanding and treating brain diseases such as Alzheimer’s. With their diversity and well-organised nervous system, polychaetes can be a new model to complement other models through systemic, cellular and molecular studies.

## Figures and Tables

**Table 1:** Polychaete species capable to regenerate anterior region

Family species	Reference
Aeolosomatidae	
*Aeolosoma* spp.	Herlant-Meewis (30)
Amphinomidae	
*Eurythoe complanata*	Muller et al. (31)
Chaetopteridae	
*Chaetopterus variopedatus*	Berrill (32)
Cirratulidae	
*Cirratulus cirratus*	Weidhase et al. (33)
*Timarete punctata*	Weidhase et al. (34)
Dorvilleidae	
*Dorvillea bermudensis*	Muller and Henning (35)
Eunicidae	
*Nematonereis unicornis*	Bely’s personal communication with Gambi MC
*Lysidice* spp.	Coulon et al. (36)
Maldanidae	
*Clymenella* spp.	Moment (37)
*Euclymene oerstedi*	Clavier (38)
*Petaloproctus socialis*	Wilson (39)
Onuphidae	
*Diopatra claparedii*	Ilmie, unpublished data
*Diopatra neapolitana*	Pires et al. (11)
Oweniidae	
*Owenia fusiformis*	Dupin et al. (40)
Phyllodocidae	
*Eulalia viridis*	Olive (41)
Sabellidae	
*Branchiomma nigromaculata*	Berrill (42)
*Myxicola aesthetica*	Berrill (43)
*Sabella* spp.	Berrill (42)
Serpulidae	
*Hydroides* spp.	Okada (44)
Spionidae	
Amphipolydora vestalis	Gibson and Paterson (45)
*Dipolydora quadrilobata*	Lindsay et al. (46)
*Pygospio elegans*	Lindsay et al. (46)
Syllidae	
*Autolytus pictus*	Okada (8)
*Procerastea halleziana*	Allen (47)
*Syllis* spp.	Verger-Bocquet (48)

**Table 2:** Comparison of well-studied animal models and polychaete in the study of nervous system

Aspects	Model organisms			

	*D. rerio*	*D. melanogaster*	*C. elegans*	Polychaete
Regeneration capability	Severed spinal cord, injured brain, damaged retina, amputated fin etc (77)	Axon, neuron of the adult, wing imaginal disk	Axon, neurons	Muscle, organs, appendages, brain, nerve cord. However, no specific studies on neuron, axon or other nerve cells regeneration available. They should be able to regenerate since both anterior and posterior can regenerate
Research focus	Alzheimer’s, genetic study, development study, cell biology	Alzheimer’s, genetic study, traumatic brain injury, regeneration study at larval stage	Learning and memory, Alzheimer’s	Taxonomy. Potentially for brain diseases such as dementia, brain injury, spinal cord injury, learning and memory
Advantage	Manipulable and transparent embryo (78)	Fully sequenced genome (79), short generation time	Transparent roundworm, short life cycle (80), 38% genes have human ortholog (81)	Progressive action of regeneration after injury, distinct separation between nervous system and other structures makes it easier to study, capable of autotomy (self-amputation), damage can be induced by amputation
Disadvantage	Complex behaviour cannot be measured, injury procedure involves dissection	No records on regeneration at adult stage, complex behaviour cannot be measured	Complex behaviour cannot be measured, no myelin, no invading macrophages, regeneration only occurs upon injury, difficult to induce injury	Still lacking of proves
